# Subclinical atherosclerosis and early brain alterations: insights from the PESA‐Brain Study

**DOI:** 10.1002/alz70856_098192

**Published:** 2025-12-24

**Authors:** Jennifer Monereo‐Sánchez, Catarina Tristão‐Pereira, Valentin Fuster, Alejandro Lopez‐Jimenez, Alberto Fernández‐Pena, Aurora Semerano, Irene Fernandez‐Nueda, Ines Garcia‐Lunar, Carmen Ayuso, Javier Sanchez‐Gonzalez, Borja Ibañez, Juan Domingo Gispert, Marta Cortes‐Canteli

**Affiliations:** ^1^ Spanish National Center for Cardiovascular Research (CNIC), Madrid, Spain; ^2^ Spanish National Center for Cardiovascular Research (CNIC), Madrid, Madrid, Spain; ^3^ Icahn School of Medicine at Mount Sinai, New York, NY, USA; ^4^ CIBER de Enfermedades Cardiovasculares (CIBERCV), Instituto de Salud Carlos III, Madrid, Spain; ^5^ University Hospital La Moraleja, Madrid, Spain, Madrid, Spain; ^6^ Health Research Institute, Fundación Jiménez Díaz University Hospital, Universidad Autónoma de Madrid, Madrid, Spain; ^7^ Philips Healthcare Iberia, Madrid, Spain; ^8^ IIS‐Fundación Jiménez Díaz, Madrid, Spain; ^9^ BarcelonaBeta Brain Research Center (BBRC), Barcelona, Spain; ^10^ Universitat Pompeu Fabra, Barcelona, Spain; ^11^ Instituto de Investigación Sanitaria Fundación Jiménez Díaz (IIS‐FJD), Madrid, Spain

## Abstract

**Background:**

Alzheimer's disease (AD) and cardiovascular disease (CVD) share overlapping risk factors and pathological pathways, yet the mechanisms linking subclinical atherosclerosis to early brain alterations remain poorly understood. The PESA‐Brain study builds on the unique strengths of the PESA cohort to explore the relationships between cardiovascular burden, cerebrovascular health, and early markers of AD pathology in asymptomatic, middle‐aged individuals.

**Method:**

PESA‐Brain is a longitudinal study that includes 1,000 participants aged 50–55 from the PESA cohort, which provides extensive cardiovascular and biomarker data collected over more than a decade. The study aims to investigate how subclinical atherosclerosis and other cardiovascular risk factors contribute to early brain alterations. Recruitment for PESA‐Brain is expected to be completed by April 2025. Current analyses focus on integrating cardiovascular metrics with multimodal data to evaluate the potential pathways linking vascular and neurodegenerative processes.

**Result:**

PESA‐Brain hypothesizes that subclinical atherosclerosis, through mechanisms such as impaired cerebral perfusion and neurovascular dysfunction, may drive early brain alterations associated with AD pathology. By identifying these pathways in midlife, the study seeks to uncover modifiable factors that can inform strategies for early prevention and intervention.

**Conclusion:**

PESA‐Brain offers a unique opportunity to understand the early interactions between cardiovascular health and brain integrity, offering critical insights into the role of vascular factors in the development of neurodegeneration and dementia. This study's findings are expected to contribute significantly to the global effort to combat Alzheimer's disease.

